# Impact of glucose levels on expression of hypha-associated secreted aspartyl proteinases in *Candida albicans*

**DOI:** 10.1186/1423-0127-21-22

**Published:** 2014-03-15

**Authors:** Leh-Miauh Buu, Yee-Chun Chen

**Affiliations:** 1 Division of Infectious Diseases, Department of Internal Medicine, National Taiwan University Hospital, No. 7, Chung-Shan South Road, Taipei 10002, Taiwan; 2 Department of Medicine, National Taiwan University, No. 7, Chung-Shan South Road, Taipei 10002, Taiwan; 3 Department of Biotechnology, National Kaohsiung Normal University, No. 62, Shenzhong Rd. Yanchao District, Kaohsiung City 82444, Taiwan

**Keywords:** *Candida albicans*, Secreted aspartyl proteinases (Saps), Glucose levels, Candidiasis

## Abstract

**Background:**

Ten secreted aspartyl proteinase (Sap) genes were identified in *Candida albicans*. The products of *SAP* genes are considered to be virulent factors of *C. albicans* that participated in causing mucocutaneous and systemic candidiasis in humans. Depending on environmental conditions, *C. albicans* may stay in yeast-form or convert into invasive hypha-form, and these issues may affect the expression of *SAP* genes. In this study we explored the component(s) of culture media that may affect the expression of hypha-associated *SAP* genes.

**Results:**

We demonstrate that glucose levels modulate both the hyphae development and the expression strength of hypha-associated *SAP* genes (*SAP4-6*). In contrast to high glucose concentration (2%), lower glucose level (0.1%) is more potent to promote hyphae development and to promptly elicit the expression of hypha-associated Sap proteins during yeast-to-hypha transition of *C. albicans*. Both Cph1-mediated MAP kinase cascade and Efg1-mediated cAMP/PKA pathway, although the latter seemed dominant, participate in convey the glucose signaling to regulate the expression of hypha-associated *SAP* genes and this glucose level effect may perform at very early stage of yeast-to-hypha transition. In addition, when *C. albicans* was co-cultured with THP-1 human monocytes, the engulfed *C. albicans* was developing hypha efficiently within 1 hr and the expression of hypha-associated Sap proteins could be detected on the distal surface of hyphae.

**Conclusion:**

We propose that the glucose level of bloodstream (approximately 0.1%) may be facilitated for stimulation of *C. albicans* to develop invasive hypha-form and to elicit promptly production of high-level hypha-associated Sap proteins.

## Background

*Candida* species are ubiquitous human commensal yeasts that reside in mucosae of oral cavity, gastrointestinal tract, female genitalia and skin. Usually they are part of a healthy individual’s normal microflora; however, in immunocompromised or disturbed ecosystem of the host, *Candida* species became pathogenic*,* causing superficial infection and/or systemic candidiasis [1-3]. The incidence of nosocomial candidiasis was increasing markedly during the last decades [4,5]. *Candida* species became the 4th most common nosocomial bloodstream isolates in United States [5]. In Taiwan, an increasing incidence of candidemia became apparent from 1980 to the end of 1990s, followed by relative stability. Crude mortality rates of patients with candidemia were in the range of 35% to 60% [6]. Among the isolates of nosocomial candidiasis, *Candida albicans* is the predominant cause of invasive candidiasis that accounts for more than 50% of all cases [5,6].

Several properties of *C. albicans* are known to contribute to its virulence those basically include the morphological transition from yeast- to hypha-form, adhesion and invasion, and secretion of hydrolytic enzymes [7]. Generally, hyphae development of *C. albicans* can be induced efficiently when they are cultured in liquid media with neutral pH and requires a temperature of 37°C [8]. Presently, ten closely related secreted aspartyl proteinase (*SAP*) genes were identified in *C. albicans*[9-13]. Studies have established the relation between secreted aspartyl proteinases (Saps) and the pathogenicity of *C. albicans*[14-17]. The mRNA expression of the *SAP4-6* subfamily was first identified in neutral pH medium with serum to induce hyphae development at 37°C [10]. Hence, the expression of *SAP4-6* was considered to be associated with the signal cascade of hyphae formation, and this may regulate by several independent but interconnected signal transduction pathways, such as cAMP/PKA pathway and MAP kinase cascade [18,19]. Moreover, hypha-associated *SAP4-6* has been investigated as the potent virulent factors in mouse model of systemic candidiasis [20,21].

Phagocytes and neutrophils constitute major obstacles to the establishment of the systemic candidiasis [7,22-24]. Sap4-6 proteins are first detected in *C. albicans* within macrophages [25]. In such situation, serum is unlikely contributing for expression of *SAP4-6* and the intracellular regulated factors remained unknown [25]. The transcriptional response of *C. albicans* upon internalization by macrophages showed that *Candida* cells underwent gluconeogenesis in the early phase to overcome the starvation [26-28]. Epidemiologic studies have shown that nothing per os and parenteral hyperalimentation are risk factors of healthcare-associated candidemia [29]. Population-based active surveillance also revealed that higher incidence of candidemia in diabetic patients [30]. In our previous study, we tried to examine the expression of hypha-associated *SAP*s at protein level. Referring to other studies, we pre-cultured the *C. albicans* in the yeast form at 25°C for 48 hr, and then subcultured the yeast form *C. albicans* in Modified Lee’s medium containing 0.2% BSA and incubated at 37°C to induce yeast-to-hypha transition. After 8 hr of filamentous growth, a little of secreted Sap5 protein began to be detected; however, the significant amount of Sap5 and trace of Sap4/6 proteins was detected after 24 hr of hyphae induction [31]. This elicited a query that how hypha-associated Sap proteins could be the potent virulent factors if they did not express promptly during the invasive process of hyphae development and need such a long period to induce their expression.

In this study, we tried to explore the component(s) of culture media that may modulate the expression of hypha-associated Sap proteins. We cultured *C. albicans* strains in several commonly used hypha-inducing media for a period at 37°C, and then inspected the hyphae development and detected the production of hypha-associated Sap proteins. By adjustment the composition of media and evaluation the effect on the filamentous growth and Saps expression, we demonstrated that glucose levels should be an important environmental factor for induction of hyphae development and modulation the expression level of hypha-associated *SAP* genes during yeast-to-hypha transition and during *C. albicans* was engulfed by phagocytes. We proposed that glucose level of bloodstream (approximately 0.1%) may be facilitated to provoke the invasive properties of *C. albicans*.

## Methods

### Strains, media, and morphological analysis

The *C. albicans* strains used in this study were listed in Table 1[20,32-35]. Media used in induction of hyphae formation and expression of hypha-associated genes included: Modified Lee’s medium [36,37] with 0.1% or 2% glucose, spider medium [38], YP medium (1% yeast extract, 2% peptone) with indicated glucose concentration. All media contained 40 mg of uridine/L to minimize the *URA3* effect [39]. For hyphae induction, *C. albicans* strains were freshly cultured on YPD (YP medium with 2% glucose) plate at 25°C or 30°C for overnight and cells were inoculated into various liquid media with initial density of OD_600_ = 1/ml, then incubated at 37°C for indicated times with gentle shaking. For observation the morphology of *C. albicans*, cells were loaded on poly-lysine coated glass slides and fixed by 3.7% formaldehyde. For each condition, 30-view of microscopy was photographed and the morphological proportion of *C. albicans* cells was calculated. The morphological analysis was defined according to the following criteria: yeast form, single cell or cell with bud; germ tube, filament </= one mother cell length; short hypha, filament </= two mother cell length; long hypha, filament > two mother cell length. Experiments were repeated three times.

**Table 1:** ***C. albicans *** **strains used in this study**

**Strain type and number**	**Genotype**	**Reference**
Clinical isolate SC5314	*URA3/URA3*	[32]
CAF4-2 (parental strain)	*ura3::imm434/ura3::imm434*	[33]
*sap2* (M12/BH52-1-17)	*sap2*::*hisG*/*sap2*::*hisG*::*URA3*::*hisG*	[34]
*sap4* (DSY436)	*sap4*::*hisG*/*sap4*::*hisG*::*URA3*::*hisG*	[20]
*sap5* (DSY452)	*sap5::hisG*/*sap5::hisG::URA3*::*hisG*	[20]
*sap6* (DSY346)	*sap6*::*hisG*/*sap6*::*hisG*::*URA3*::*hisG*	[20]
*efg1*	*efg1*::*hisG/efg1*:: *hisG*::*URA3*::*hisG*	[35]
*cph1*	*cph1*::*hisG*/*cph1*::*hisG*::*URA3*::*hisG*	[35]

### Co-culture of *C. albicans* with THP-1 human monocytes

The THP-1 human monocytic cell line [40] was maintained in RPMI1640 with 10% fetal bovine serum (RPMI-FBS) at 37°C in a humidified chamber containing 5% CO_2_. For co-culture, THP-1 cells were cultured in the 10-cm dishes for 2 days, then removed medium and suspended cells in fresh RPMI-FBS by gentle pipetting and incubated at 37°C for 10 min before co-cultured with *C. albicans*. About 2 × 10^6^*C. albicans* cells were co-cultured with 2×10^5^ THP-1 cells in 1.5 ml of RPMI-FBS in a 2 ml microcentrifuge tube at 37°C incubator for indicated times with gentle rocking [40].

### Protein isolation and Western blotting

Proteins that existed in culture media were precipitated by 10% TCA (trichloroacetic acid). For investigation on the expression of hypha-associated Sap proteins in *C. albicans* during phagocytosis, *C. albicans* SC5314 was co-cultured with THP-1 cells. After 1 hr cultivation at 37°C, co-cultured cells were harvested and suspended in lytic solution (Tris–HCl, pH7.5, 10 mM; EDTA 10 mM; NaCl 50 mM; SDS 0.2%). Then ddH_2_O was added to thoroughly lyse the THP-1 cells. *Candida* cells were harvested by low speed centrifugation (1,200 g for 3 min) and were washed once by ddH_2_O. Then *Candida* cells were suspended in solution containing 1% β-mercaptoethanol and Zymolase. After incubating at 37°C for 30 min, the secreted proteins that were released in supernatant were precipitated by 10% TCA. The Western blotting procedure and preparation of polyclonal anti-Sap6 antibody have been described [31].

### Immunofluorescence of hypha-associated Sap proteins

*C. albicans* and THP-1 cells were co-cultured at 37°C for 30 min (for phagocytosis), then cells were harvested by low speed centrifugation and re-suspended in RPMI-FBS and incubated at 37°C for further 30 min (hyphae development of THP-1-engulfed *Candida* cells). Co-cultured cells were harvested and suspended in PBS and loaded on poly-lysine coated cover glasses. The coated cells were fixed by 3.7% of formaldehyde in PBS for 15 min and were permeated by 0.2% TritonX100 for 3 min. After blocking (PBS + 5% skim milk), cells were incubated with anti-Sap6 antibody (1:500-dilution) for 90 min. The detailed procedure has been described [41].

### RNA preparation and reverse transcription-polymerase chain reaction

*C. albicans* SC5314 (OD_600_ = 10) was inoculated into YPD, RPMI-FBS, or co-cultured with THP-1 cells in RPMI-FBS, respectively, then incubated at 25°C for YPD and at 37°C for later two media. *Candida* cells were harvested after 30 min cultivation and total RNA was isolated by hot acid phenol method [42]. Before reverse transcription, 2 μg of total RNA was treated by DNaseI (Invitrogen). The SuperScriptIII (Invitrogen) and oligo-(dT)_12–18_ were used to generate the cDNA. The expression of hypha-associated *SAP* genes was identified by PCR amplification using specific primers that were listed in Table 2[19].

**Table 2:** Primers used for reverse transcription-PCR analysis in this study

**Primer**	**Sequence (5′ to 3′)**	**Reference**
SAP4-1	TGCCGATGGTTCTGTTGCAC	This study
SAP4-2	AACTTGAGCCATGGAGATCTTTC	This study
SAP5-1	GCGGCGAAGCTACCGAGTTTG	This study
SAP5-2	TACCACTAGTGTAATATGTTTGGA	This study
SAP6a	AAACCAACGAAGCTACCAGAAC	[19]
SAP6b	TAACTTGAGCCATGGAGATTTTC	[19]
EFB1a	AGTCATTGAACGAATTCTTGGCTG	[19]
EFB1b	TTCTTCAACAGCAGCTTGTAAGTC	[19]

## Results

### Expression of Sap5 in hypha-inducing conditions

**Figure 1:**
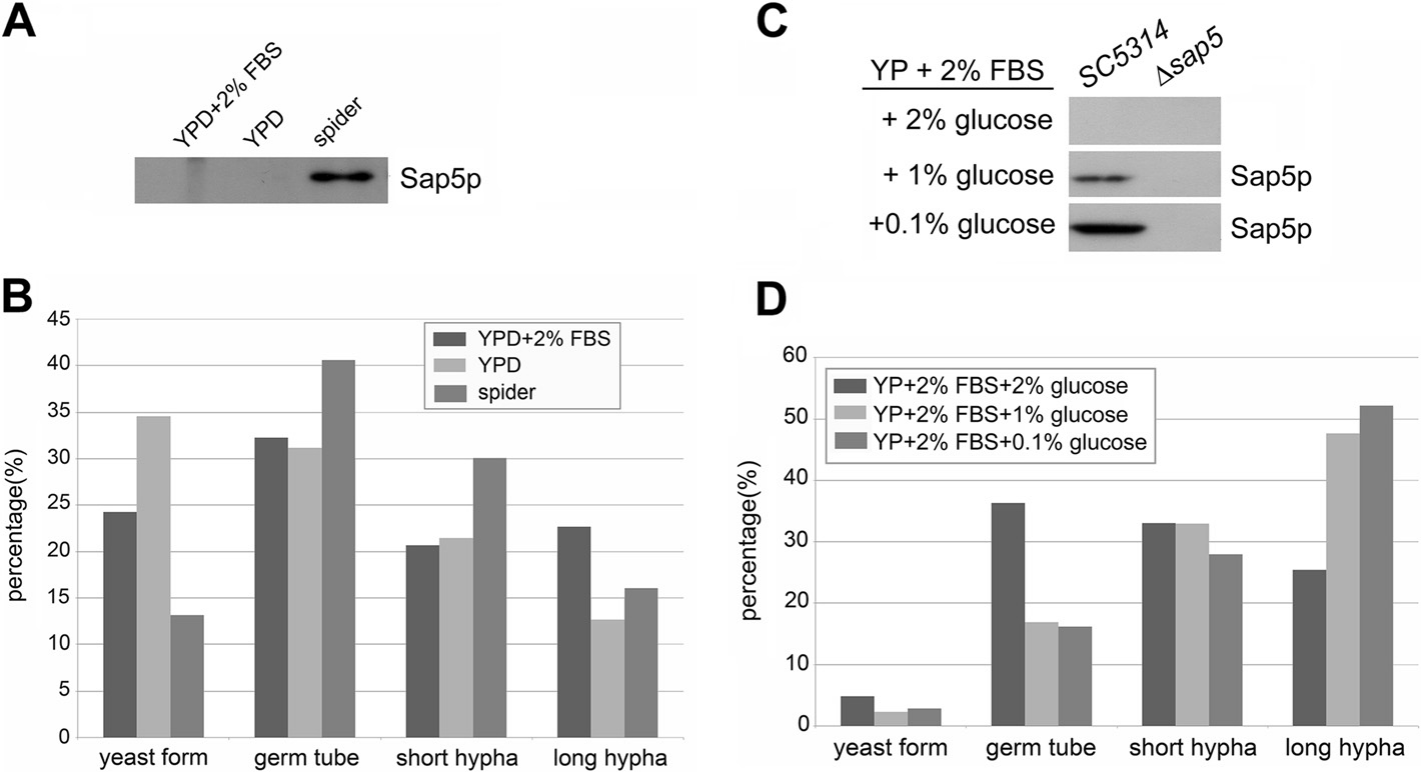
**Expression of Sap5 in spider medium and lower glucose level media. ***C. albicans* SC5314 was inoculated into various media with initial density of OD_600_ = 1/ml. Cells were harvested after cultured at 37°C for 1 hr. The media components were precipitated by 10% TCA for Western blot analysis and the cells were fixed by 3.7% formaldehyde for morphology observation. **(A)** The secreted Sap5 (Sap5p) was detected in precipitate of spider medium by Western blotting using polyclonal anti-Sap6 antibody which can recognize Sap4 ~ 6 proteins. **(B)** The analysis of morphological proportion of *C. albicans* SC5314 that was cultured in panel **(A)** described condition. **(C)** The Sap5 was rapidly and highly expressed in low glucose concentration (0.1%) during yeast-to-hypha transition. FBS: fetal bovine serum. **(D)** The analysis of morphological proportion of *C. albicans* SC5314 that was cultured in panel **(C)** described condition.

For explore the factor(s) that may have an effect on the expression of Sap proteins, we firstly examined whether the hypha-associated Sap proteins can be expressed in commonly used hypha-induced media. After several times of pre-test, we incubated *C. albicans* SC5314 at 37°C for 1 hr in different liquid media with initial cell density of OD_600_ = 1/ml to induce hyphae development. This culture condition was applied in all of hypha-inducing experiments of this study. The expression of hypha-associated Sap4 ~ 6 proteins were identified by Western blotting using polyclonal anti-Sap6 antibody which can recognize Sap4 ~ 6 proteins [31]. Moreover, the morphology of *Candida* cells was inspected and calculated. The results demonstrated that about 85% of *Candida* cells undertook yeast-to-hypha transition (germ tube + short hypha + long hypha) when cultured in spider medium, and 75% in YPD medium containing 2% fetal bovine serum (FBS), and 65% in YPD medium (Figure 1B). Secreted Sap5 protein, which is the most abundant secreted Sap protein during hyphae development, was only detected in precipitates of spider medium (Figure 1A) that contained mannitol instead of glucose. This result provide a hint that hyphae development of *C. albicans* can be induced efficiently under 37°C in almost all commonly used neutral pH media, but the expression rate and expression level of hypha-associated Sap proteins may be affected by certain components of culture media. By comparison the media compositions we suspected the glucose level, but not the addition of serum or BSA that was commonly used in many studies, may be an important issue to affect the expression of Sap proteins in the course of yeast-to-hypha transition.

### Glucose levels modulate Sap5 expression in hypha-inducing conditions

In order to elucidate the glucose effect on the promptly expression of Sap proteins during hyphae development, we cultivated *Candida* cells in YP medium containing 2% FBS and adding 2%, 1%, or 0.1% of glucose, respectively, at 37°C for 1 hr. The results demonstrated that in these culture conditions, more than 95% of *Candida* cells underwent yeast-to-hypha transition. Indeed, *Candida* cells that cultured in 1% and 0.1% glucose exhibited similar ratio of yeast-to-hypha transition (Figure 1D), but secreted Sap5 protein was far more abundant in medium containing 0.1% glucose (Figure 1C). These results demonstrated that hyphae development of *C. albicans* can be enhanced by serum supplement, but the glucose levels display a substantial effect on regulation the expression strength of hypha-associated Sap5 protein.

### Low glucose level enhances yeast-to-hypha transition and provokes Saps expression

**Figure 2:**
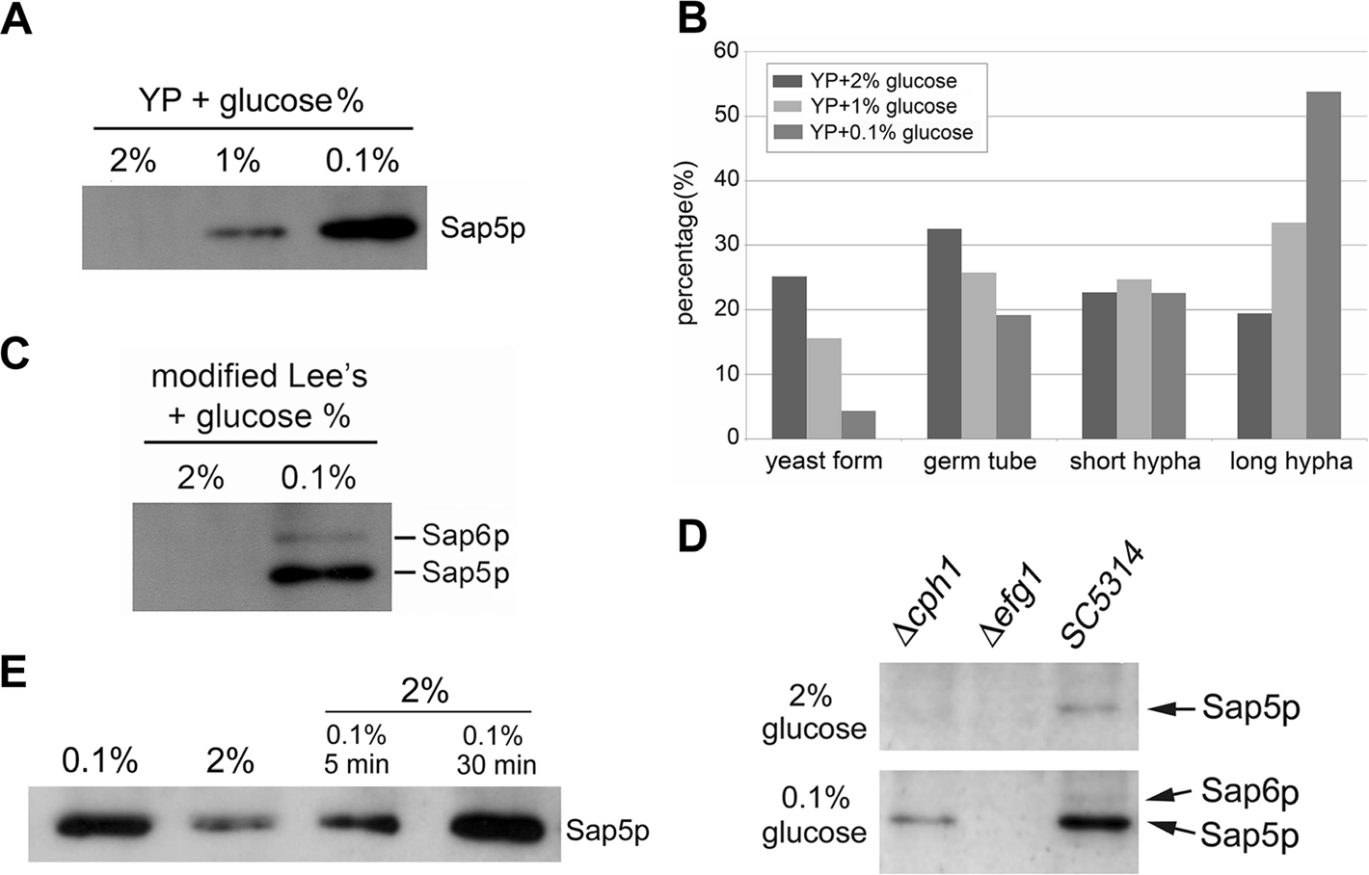
**Low glucose level enhances hyphae formation and provokes the expression of hypha-associated Sap proteins. (A) ***C. albicans* SC5314 was cultured at 37°C for 1 hr in YP medium containing different concentration of glucose. More of secreted Sap5 was detected in medium with lower glucose concentration (0.1%). **(B)** The analysis of morphological proportion of *C. albicans* SC5314 that was cultured in panel **(A)** described condition. **(C)** Abundant Sap5 and trace of Sap6 proteins were detected in Modified Lee’s medium containing 0.1% glucose after 2 hr incubation at 37°C. **(D)** The expression of Sap5 and Sap6 was impaired in *cph1*-null strain and was abolished in *efg1*-null strain. **(E)** Exploration the high glucose level represses Sap5 expression during yeast-to-hypha transition. *C. albicans* SC5314 was cultured in YP medium containing 0.1% glucose at 37°C. Glucose level was re-added up to 2% at indicated incubation times and then still cultured at 37°C; the total incubation period was 90 min. Secreted Sap5 was detected by Western blot analysis.

Above results also demonstrated that high glucose level seems to delay the hyphae expansion of *C. albicans* even though in the serum supplement condition (Figure 1D). We further investigated whether the glucose levels affect the hyphae formation and the expression of hypha-associated Sap proteins. The results showed that without serum, after 1 hr incubation at 37°C, lower glucose level (YP + 0.1% glucose) could induce hyphae formation more efficiently (Figure 2B), and more of secreted Sap5 was identified in medium under this condition (Figure 2A). Hence, low glucose level facilitates hyphae development and rapidly enhances Sap5 expression during the yeast-to-hypha transition.

Moreover, we incubated *C. albicans* strains in synthetic Modified Lee’s medium containing 2% or 0.1% glucose, respectively, and without BSA supplement. After 2 hr cultured at 37°C, abundant Sap5 and trace of Sap6 (compared wild type strain with *sap*-deleted mutants, data not shown) were detected in medium with 0.1% glucose (Figure 2C). Again, this result proved that the glucose level has an effect on modulation the expression level of hypha-associated Sap proteins. Moreover, this result can explain our previous study [31] that *C. albicans* after 12 hr of filamentous growth at 37°C in Modified Lee’s medium containing 0.2% BSA and 2% glucose, the glucose was consumed gradually, more Sap5 and even Sap6 could be detected in lower glucose concentration medium.

### Regulation the expression of hypha-associated Sap proteins in hyphae development with low glucose condition

Efg1 and Cph1 are respective the downstream effector of cAMP/PKA signaling pathway and MAP kinase cascade, and both were shown to regulate the hyphae development and the expression of hypha-associated virulent factors in *C. albicans* including hypha-associated Sap proteins [19,43]. When cultivation of *C. albicans* strains in Modified Lee’s medium containing 2% glucose at 37°C for 4 hr, the expression of Sap proteins was undetected in *efg1*-null or *cph1*-null mutants and only trace of Sap5 protein could be detected in wild type SC5314 strain. However, when *C. albicans* strains were cultured in 0.1% glucose at 37°C for 4 hr, abundant Sap5 protein and little of Sap6 protein were produced by wild type strain SC5314 and trace of secreted Sap5 protein could be detected in medium of *cph1*-null mutant (Figure 2D). In fact in liquid media, the *cph1*-null strain appeared delayed hyphae development, and the filamentous growth of *efg1*-null mutant was abolished (data not shown). Hence, we deduced that both cAMP/PKA signaling pathway and the MAP kinase cascade can convey the glucose signaling that may sequential or cross-connect to affect the yeast-to-hypha transition and modulate the expression of hypha-associated Sap proteins, especially the Efg1-mediated cAMP/PKA pathway dominates the major effect.

Besides, we also investigated the suppressive effect of high glucose level on Sap5 expression. We cultured *C. albicans* cells in YP medium containing 0.1% glucose at 37°C and glucose concentration was re-added up to 2% at several incubation time points respectively, and then still cultured at 37°C; the total incubation period was 90 min. The Western blotting revealed (Figure 2E) the Sap5 expression can be repressed when glucose concentration was re-added up to 2% at the time point of 5 min incubation. However, after 30 min incubation, the Sap5 expression was not repressed effectively by replenished high glucose concentration. In fact, even as early as re-added glucose concentration up to 2% at 10 min incubation, the suppressive effect of high glucose level on Sap5 expression was not evident (data not shown). Therefore, the glucose level modulates the expression of hypha-associated *SAPs* may perform at very early stage during yeast-to-hypha transition of *C. albicans*.

### Expression of hypha-associated Sap proteins during phagocytosis

**Figure 3:**
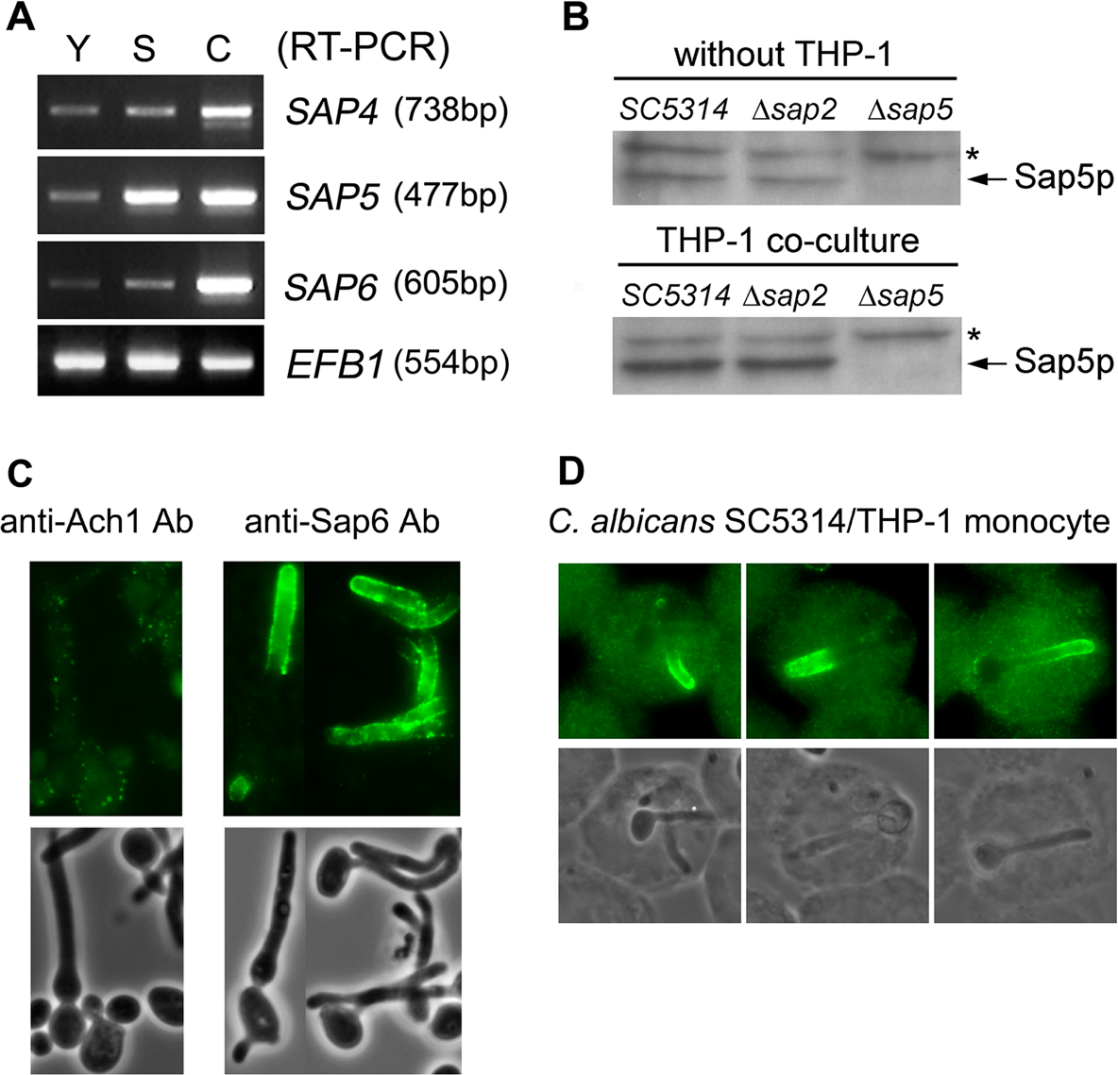
**Expression of hypha-associated Sap proteins during phagocytosis. (A) ***C. albicans* SC5314 was cultured in YPD medium at 30°C to maintain yeast form, in RPMI-FBS (which contained 10% FBS and 0.4% glucose) medium at 37°C to generate hypha-form, and co-cultured *C. albicans* with THP-1 human monocytes in RPMI-FBS medium at 37°C. The expression of hypha-associated *SAP* genes was identified by reverse transcription-PCR analysis using gene specific primers. Y: YPD, S: RPMI-FBS, C: co-culture. *EFB1* gene is an internal control. **(B)** Detection the secreted Sap5 protein in *C. albicans* and THP-1 co-culture condition by Western blot analysis. The * is a non-specific signal. Protein samples of the upper panel and the lower panel were manipulated simultaneously. **(C)** Detection the secreted Sap proteins on hyphae surfaces of *C. albicans* by immunofluorescence staining using polyclonal anti-Sap6 antibody as primary antibody. The polyclonal anti-Ach1 antibody is a negative control. **(D)** Detection the expression of secreted hypha-associated Sap proteins during phagocytosis by immunofluorescence staining. The majority of fluorescent signals distributed on the distal surface of hyphae.

We also examined the expression of hypha-associated Sap proteins in co-culture system of THP-1 human monocyte and *C. albicans*. The composition of THP-1 culture medium (RPMI1640 with 10% FBS) contained about 0.4% glucose. Reverse transcription-PCR analysis revealed that the mRNA level of hypha-associated *SAP* genes (*SAP4 ~ 6*) was elevated after 30 min incubation of *C. albicans* in cell culture medium (RPMI-FBS) at 37°C (Figure 3A, compare Y and S). Moreover, the expression of *SAP4 ~ 6* genes was further enhanced in co-cultured condition (Figure 3A, compare S and C), and this result was proved by Western blotting (Figure 3B). Besides, after 1 hr co-culture at 37°C, the engulfed *C. albicans* was developing hyphae efficiently in THP-1 cells, and the immunofluorescence showed that hypha-associated Sap proteins were detected on the distal surface of hyphae (Figure 3C, D). These results demonstrated that *C. albicans* could develop filamentous growth rapidly after phagocytosis and considerable amount of hypha-associated Sap proteins would be expressed promptly during this process.

## Discussions

This study manifested that during hyphae development of *C. albicans*, the expression strength of hypha-associated Sap proteins can be modulated by glucose levels. Serum is known to be an efficient substance to enhance yeast-to-hypha transition of *C. albicans* at 37°C, but it seems not to be the most potent factor to elicit the expression of hypha-associated Sap proteins during hyphae development [44]. Yeasts have been shown to have a refined genetic program that responsible for sensing, acquisition, and utilization of glucose [45,46]. In the in vitro environment, glucose depletion can cause invasive growth of *S. cerevisiae* on solid agar [47]. In addition, filamentous growth of *C. albicans* can be induced effectively on solid minimal medium containing 0.1% glucose relative to higher glucose levels [48]. In our study of culture conditions, low glucose level (0.1% glucose) is more potent to promote hyphae formation and to elicit the expression of *SAP5* and *SAP6* accompanying with yeast-to-hypha transition of *C. albicans*, and the protein products can be detected after short period (~1 hr) of hyphae induction.

Although glucose is abundant and convenient for utilizing on earth, it is usually a restrictive nutrient within biological systems for commensal and parasitical microorganisms. Therefore, it is reasonable to consider that the natural habitats of *C. albicans* should be glucose-limited. In both *S. cerevisiae* and *C. albicans*, three independent but cross-regulated signal transduction pathways have been shown to sense and convey the glucose signaling and eventually to generate a coordinated response [49]. The Hgt4 of *C. albicans* is presently known as a high-affinity glucose sensor which can respond to glucose levels as low as 0.01% [50]; certainly, this sensor is considered to be functional when *C. albicans* survived under physiological glucose levels of human host (0.1%). In addition, activated Gpr1/Gpa2-GTP and activated Ras1-GTP may also function as glucose-sensing system that to modulate cAMP/PKA pathway [49,51]. However, abundant glucose may elicit the glucose repression pathway that enable to repress genes required for the usage of nonfermentable carbon sources, genes in the process of the cAMP/PKA pathway, and genes encoded the high-affinity Hgt proteins which required in glucose-limited condition [49]. Accordingly, we assumed that the glucose level in physiological condition of human is sufficient to activate the glucose utilization network in *C. albicans* but not high enough to provoke the glucose repression response. A recent study indicated that glucose levels in the bloodstream (0.05-0.1%) have a crucial effect upon the gene regulation of *C. albicans*[52]. However, most of researchers cultured *C. albicans* in yeast extract-peptone-dextrose (YPD) or other media, such as synthetic complete medium, usually containing 2% glucose and provided serum to enhance hyphae development at 37°C. In these culture conditions, expression of many genes may be repressed by high glucose level and the resulted profiles of gene expression may far different from the state of *C. albicans* in its in vivo habitats. Besides, earlier studies have demonstrated that low glucose concentration (0.1%) could provoke the germ-tube formation of *C. albicans* more efficiently than that of high glucose levels [53,54]. Indeed, in our study, expression of Sap5 and Sap6 also could be rapidly induced when cultured *C. albicans* at 37°C in YP (yeast extract-peptone) medium without glucose containing (data not shown). Hence, glucose concentration in culture media should be considered as an important modulation factor when studies the gene expression patterns of *C. albicans*.

Moreover, our results demonstrated both cAMP/PKA pathway and the MAP kinase cascade in *C. albicans* are important for regulation the high expression level of hypha-associated Sap proteins that triggered by 0.1% glucose, especially through the Efg1-mediated cAMP/PKA pathway. Studies have revealed that some environmental signals, including nitrogen and glucose levels, can be conveyed to both cAMP/PKA and the MAP kinase pathways through regulator Ras1 [8,51]. However, the Efg1 is the dominant effector for hyphae formation when *C. albicans* was cultured in liquid media, and the cAMP/PKA pathway plays major role in initiation of hyphae development under most conditions [8]. Therefore, we suggested that prompt expression of hypha-associated Sap proteins may depend on efficient initiation of hyphae development by Efg1-mediated cAMP/PKA pathway and the maintenance of high expression level of hypha-associated Sap proteins may require the coordination of Cph1-mediated MAP kinase cascade.

Several host defense proteins in the mucosa surface, such as salivary lactoferrin, α-macroglobulin (proteinase inhibitor), secretory immunoglobulin A, and enzymes of the respiratory burst of macrophages can be hydrolysed by Sap proteins [55]. These macrophages are predominantly located beneath the intestine epithelial layer within the lamina propria [56-58]. Based on our in vitro studies, we inferred that during in vivo infection, once *C. albicans* transferred from intestinal or other mucosa surfaces to invade deep tissues where temperature near 37°C, and/or intravascular invasion facing glucose concentration around 100 mg/dL (0.1%) [48]; both signals and serum would efficiently induce hyphae formation and elicit expression of hypha-associated Sap proteins in *C. albicans*. In addition, the study of transcriptional response has revealed that upon internalization by macrophages, the *C. albicans* underwent gluconeogenesis in the early phase to overcome the starvation situation [27]. Besides, inside the phagocytes, the phagosome-containing microorganisms would fuse with cellular lysosomes to form the microbicidal phagolysosomes; however, many pathogenic microbes are able to endure in phagocytes that may render by activating the potent virulent factors of microbes during phagocytosis [25]. In our study, we demonstrated that the rapidly enhanced expression of hypha-associated *SAP* genes was one of the downstream responses which were modulated by factors activating by the stress and starvation signals after *C. albicans* was engulfed in phagocytes.

## Conclusions

This study demonstrated that adjusting the glucose levels in culture media to imitate the physiological condition of human (0.1% glucose) is efficient to enhance the expression level of hypha-associated *SAP* genes in *C. albicans*, and this condition may also regulate the expression of other virulent factors during hyphae development of *C. albicans*. In addition, this study provides biological evidence for the clinical findings that nothing per os, parenteral hyperalimentation and poor controlled diabetes, which may influence the in vivo glucose levels, are risk factors of candidiasis.

## Abbreviations

SAP: Secreted aspartyl proteinase; BSA: Bovine serum albumin; FBS: Fetal bovine serum; TCA: Trichloroacetic acid; dL: Deciliter.

## Competing interests

The authors declare that they have no competing interests.

## Authors’ contributions

LMB designed and manipulated the experiments and participated in manuscript writing. YCC discussed the experimental design and results with LMB and participated in manuscript writing. Both authors read and approved the final manuscript.
